# RNA-Seq analysis of *Citrus reticulata* in the early stages of *Xylella fastidiosa* infection reveals auxin-related genes as a defense response

**DOI:** 10.1186/1471-2164-14-676

**Published:** 2013-10-03

**Authors:** Carolina M Rodrigues, Alessandra A de Souza, Marco A Takita, Luciano T Kishi, Marcos A Machado

**Affiliations:** 1 Departamento de Biotecnologia, Centro APTA Citros Sylvio Moreira, CP4, Cordeirópolis, SP 13490-970, Brazil; 2 Departamento de Genética e Evolução, Universidade Federal de São Carlos UFSCAR, CP676, São Carlos, SP 13565-905, Brazil

**Keywords:** Gene expression, CVC, Plant-pathogen interaction, Ponkan mandarin, Pera sweet orange, Resistance

## Abstract

**Background:**

Citrus variegated chlorosis (CVC), caused by *Xylella fastidiosa*, is one the most important citrus diseases, and affects all varieties of sweet orange (*Citrus sinensis* L. Osb). On the other hand, among the Citrus genus there are different sources of resistance against *X. fastidiosa*. For these species identifying these defense genes could be an important step towards obtaining sweet orange resistant varieties through breeding or genetic engineering. To assess these genes we made use of mandarin (*C. reticulata* Blanco) that is known to be resistant to CVC and shares agronomical characteristics with sweet orange. Thus, we investigated the gene expression in Ponkan mandarin at one day after infection with *X. fastidiosa*, using RNA-seq. A set of genes considered key elements in the resistance was used to confirm its regulation in mandarin compared with the susceptible sweet orange.

**Results:**

Gene expression analysis of mock inoculated and infected tissues of Ponkan mandarin identified 667 transcripts repressed and 724 significantly induced in the later. Among the induced transcripts, we identified genes encoding proteins similar to Pattern Recognition Receptors. Furthermore, many genes involved in secondary metabolism, biosynthesis and cell wall modification were upregulated as well as in synthesis of abscisic acid, jasmonic acid and auxin.

**Conclusions:**

This work demonstrated that the defense response to the perception of bacteria involves cell wall modification and activation of hormone pathways, which probably lead to the induction of other defense-related genes. We also hypothesized the induction of auxin-related genes indicates that resistant plants initially recognize *X. fastidiosa* as a necrotrophic pathogen.

## Background

The Brazilian citrus industry accounts for 30% of sweet orange production and 85% of exports of frozen-concentrated orange juice in the world, despite the large number of pests and diseases that affect the Brazilian orchards. Among these diseases, Citrus Variegated Chlorosis (CVC), caused by the bacterium *Xylella fastidiosa*, costs around 120 million US dollars a year to chemically control the bacterial vectors and for replanting new orchards [1].

The symptoms of this disease are associated with the blockage of xylem vessels by *X. fastidiosa* biofilm, leading to increased water stress and decreased nutrients in the diseased plant [2-4].

*Citrus* species show varying responses to CVC. While the sweet orange (*Citrus sinensis* L. Osb) is very susceptible, the Ponkan mandarin (*Citrus reticulata* Blanco) is considered resistant because it shows no symptoms, yet the bacteria can be isolated from the plants at 30 days after inoculation. However, after 60 days of inoculation the bacteria cannot be isolated from the plant. The resistance of mandarin is not related to the number and/or diameter of xylem vessels, suggesting that resistance is caused by active defense responses [5]. Based on this, the pattern of gene expression in Ponkan mandarin was assessed by sequencing expressed sequence tags in mandarins inoculated with *X. fastidiosa* at 30 and 60 days after infection. The results revealed differential expression patterns for several defense-related genes of the salicylic acid (SA), jasmonate (JA), and ethylene (ET) signaling pathways [6,7]. These results indicate a crosstalk between regulatory pathways that control different cellular processes in the mandarin-*X. fastidiosa* interaction. However, it is unclear whether these pathways are activated during the initial response of Ponkan mandarin to this phytopathogen. Thus, the present study aimed to evaluate which genes are activated in the preliminary stages of infection, as this phase may involve an important strategy for avoiding pathogen establishment and colonization, and consequently the progress of the disease. Identifying these defense genes could be an important step towards obtaining sweet orange resistant varieties through breeding or genetic engineering.

## Results and discussion

### Overview of RNA-seq analysis

In recent years the number of works using global expression analysis to study plant-pathogen interactions has grown considerably. By comparing specific mRNAs present in different tissues, such as infected or not infected, differentially expressed genes can be identified and their functions inferred.

In the present study, we used RNA-seq to analyze the differential expression of Ponkan mandarin mRNAs one day after *X. fastidiosa* infection (compared with mock inoculated plants). The presence or absence of bacteria in the plants used in this analysis was confirmed by real-time quantitative PCR (RT-qPCR) (Additional file 1). Three biological replicates for each condition were selected for performing transcriptome analyses.

RNA-seq generated 35,344,265 and 37,326,339 single end reads of 101 bp for the non-infected and infected libraries, respectively. Tophat was used to align the reads to the *Citrus clementina* reference genome, with approximately 74% of success. The expression level of 27,889 coding sequences was quantified using the software Cufflink. A total of 1,391 transcripts showed significant variation in expression by Cuffdiff analysis: 724 were induced and 667 were repressed (*P* ≤ 0.001) in the infected Ponkan mandarin (Additional file 2).

**Figure 1:**
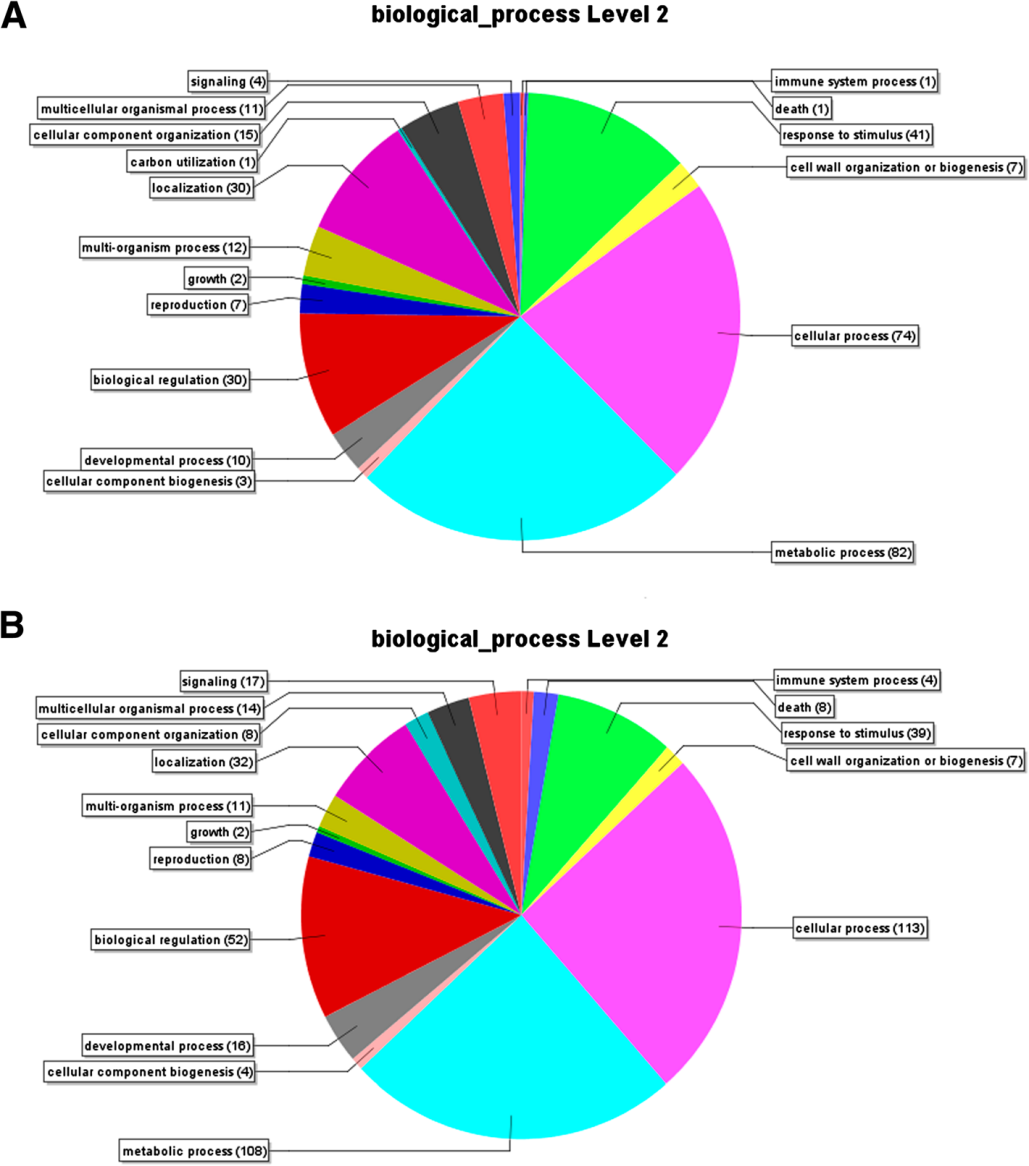
**Categorization of genes induced or repressed in Ponkan mandarin infected with *X. fastidiosa*.** The genes were automatically classified based on GO (Gene Ontology) classifications and are grouped according to their functions in plant cells. **A**. Induced genes. **B**. Repressed genes.

The differentially expressed genes were categorized using Gene Ontology (GO). Based on similarity, transcripts were distributed into different categories of biological processes (level 2). The prominent functional categories for both induced and repressed genes were related to metabolic process, followed by cellular process, response stimulus, biological regulation, and localization (Figure 1A and B). The majority of induced genes are involved in the formation of secondary xylem, cell wall, lignin, hormone synthesis, and detoxification. On the other hand, the repressed genes are related to growth, cell wall degradation, cell wall loosening, cell differentiation, and development. A complete list of *X. fastidiosa*-modulated pathways for Ponkan mandarin data sets is shown in Additional file 2.

### Induction of a PAMP - Triggered Immunity (PTI) – like response in Ponkan mandarin

In plants, Pattern Recognition Receptors (PRRs) perform the first level of microbial recognition, perceiving molecular signatures characteristic of a whole class of microbes, which are termed Pathogen-Associated (or Microbe-Associated) Molecular Patterns (PAMPs or MAMPs) [8]. PAMP recognition leads to a chain of signaling events broadly referred to as general defense responses in plants [9]. Stimulation of PRRs is a key step in the early stages of PTI. We identified two upregulated genes (Ciclev10004108m and Ciclev10014130m), which are similar to a leucine-rich repeat receptor-like protein kinase (LRR-RLK) in *Arabidopsis* (Table 1), belonging to the LRR-XII subfamily, of which EF-Tu and Flagellin sensing 2 receptors are members [10]. Upregulation of these genes suggests that PAMPs from *X. fastidiosa* might be perceived by PRRs in Ponkan mandarin and could trigger the PTI response. After this recognition, the plant modulates the expression of defense genes as well as initiating metabolic rearrangements, and ultimately activates basal resistance to potential pathogens [11]. Another upregulated gene in Ponkan mandarin encodes a leucine-rich repeat receptor-like protein (RLP12), which is associated with different functions such as cell differentiation, plant growth, development, and mainly disease resistance [12-14] (Table 1).

**Table 1:** **Differentially expressed genes in Ponkan mandarin in response to infection by *X. fastidiosa***

**Gene symbol**	gene id_Citrus clementina*	AGI**	Fold change***	**P ≤ 0.001**	**Gene description**
LRR-RLK	Ciclev10004108m	AT4G08850	1.05611	0.000755101	leucine-rich repeat family protein
LRR-RLK	Ciclev10014130m	AT3G47570	2.64522	3.84525e-09	leucine-rich repeat protein kinase family protein
RLP12	Ciclev10003540m	AT1G71400	1.20144	2.69007e-06	receptor like protein 12
CC-NBS-LRR	Ciclev10007304m	AT4G27190	1.29361	4.28293e-06	nb-arc domain-containing disease resistance protein
MYB domain	Ciclev10012089m	AT2G37630	1.35348	0.000930383	as1 (asymmetric leaves 1); transcription factor
MYO	Ciclev10010780m	AT4G33200	2.76714	0.000191443	myosin, putative
MYB66	Ciclev10017556m	AT5G14750	1.05408	1.04467e-07	myb domain protein 66); transcription factor
PAL	Ciclev10030821m	AT2G37040	0.91583	2.53552e-07	phenylalanine ammonia-lyase
GSL07	Ciclev10030560m	AT1G06490	1.0981	2.36835e-05	glucan synthase-like 7
CSLA09	Ciclev10031284m	AT5G03760	1.33401	1.33936e-07	cellulose synthase like A9
CESA8	Ciclev10014155m	AT4G18780	1.27949	6.74384e-08	cellulose synthase
CESA4	Ciclev10018639m	AT5G44030	1.15575	7.36883e-05	cellulose synthase
ATFXG1	Ciclev10031741m	AT1G67830	-0.940316	1.02739e-05	alpha-L-fucosidase/ carboxylesterase
#	Ciclev10020423m	AT4G24780	-1.03748	3.74844e-06	pectate lyase family protein
#	Ciclev10030947m	AT1G70370	-1.27505	1.32952e-06	BURP domain-containing protei
QRT2	Ciclev10005317m	AT3G07970	-1.57918	4.63421e-06	QRT2; polygalacturonase
#	Ciclev10004783m	AT1G04680	-1.68649	1.5906e-05	pectate lyase family protein
LOX	Ciclev10014202m	AT3G45140	0.99318	0.000466003	lipoxygenase 2
NCED6	Ciclev10006710m	AT3G24220	2.69285	1.90022e-09	nine-cis-epoxycarotenoid dioxygenase 6
CCD7	Ciclev10027500m	AT2G44990	0.93434	0.00201556	carotenoid clevage dioxygenase 7
AP2	Ciclev10010403m	AT1G51120	2.21376	5.89723e-07	ap2 domain-containing transcription factor, putative
AIP2	Ciclev10008969m	AT5G20910	-1.33947	4.99589e-07	zinc finger (c3hc4-type ring finger) family protein
IAA9	Ciclev10025873m	AT5G65670	2.26201	0.0033	indole-3-acetic acid inducible 9
E3 RING	Ciclev10005027m	AT2G30580	1.46874	1.05377e-05	ubiquitin-protein ligase
HECT (UPL5)	Ciclev10014213m	AT4G12570	1.41737	6.03935e-06	ubiquitin protein ligase 5
E3/SCF/FBOX (LKP1)	Ciclev10007762m	AT5G57360	0.98971	0.00243897	ubiquitin-protein ligase
ARF-GAP DOMAIN 8	Ciclev10011983m	AT4G17890	0.87335	0.00250141	arf gtpase activator
ARF8	Ciclev10014200m	AT5G37020	0.83349	0.00155566	transcription factor
ARF19	Ciclev10007286m	AT1G19220	1.17169	0.000240451	transcription factor
BIG	Ciclev10010885m	AT3G02260	0.62707	0.000252397	auxin transport protein (big)
XTH16	Ciclev10005561m	AT3G23730	-0,983574	5.04445e-07	xyloglucan endotransglycosylase, putative
EXGT-A4	Ciclev10028851m	AT5G13870	-1,37398	5.49438e-11	engoxyloglucan transferase A4
ATEXPA4	Ciclev10012518m	AT2G39700	-1,99214	0.000463125	expansin A4

* *Citrus clementina* transcripts identification number (reference genome used for mapping the reads) - http://www.phytozome.org/search.php.

** Identification number of the *Arabidopsis thaliana* ortholog of up and down-regulated citrus gene in response to *X. fastidiosa* infection (The Arabidopsis Genome Initiative).

*** Log_2_ fold change values (P ≤ 0.001) obtained from of each infected sample compared to mock-inoculated control.

# unnamed gene symbol.

Up and downregulated genes in Ponkan mandarin 24 h after infection with *X. fastidiosa* compared with the control.

A gene encoding a coiled-coil motif nucleotide-binding site–leucine-rich repeat (CC-NBS-LRR) protein was upregulated in Ponkan mandarin challenged with *X. fastidiosa* (Table 1). CC-NBS-LRR is a cytoplasmic receptor normally involved in responses triggered after recognition of Avr proteins secreted by the pathogen. However, this bacterium does not have the type III secretion apparatus or Avr proteins [15]. This leads us to believe that this receptor may be involved in perception of damage-associated molecular patterns (DAMPs), because the recognition of cytoplasmic danger signals depends on cytoplasmic sensors like NB-LRR resistance proteins [16-18]. This hypothesis is consistent with *X. fastidiosa*’s ability to produce danger molecules by degrading plant cell walls [4]. However, this hypothesis needs to be further investigated.

A main feature of the PTI response is strengthening of the cell wall [19]. Many genes related with cell wall modification were upregulated in Ponkan mandarin infected with *X. fastidiosa*, which reinforced our hypothesis that PTI is involved in this early response.

### Genes related to secondary metabolism and the cell wall

**Figure 2:**
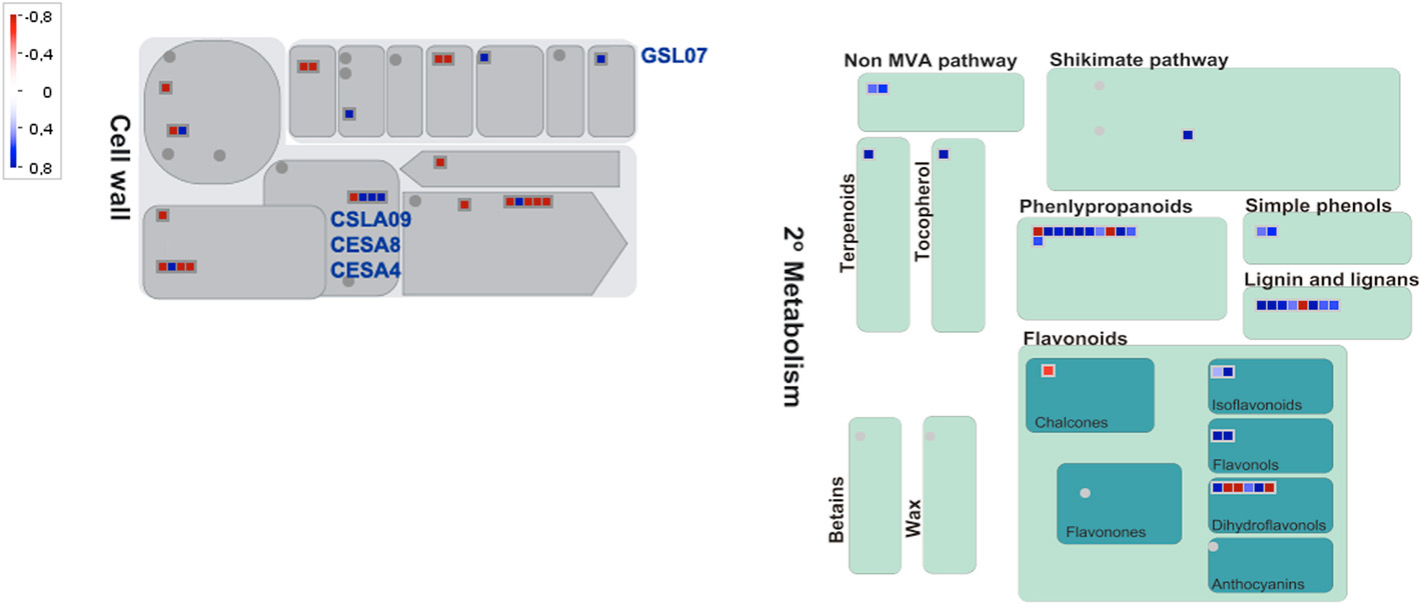
**Responses related to secondary metabolism and the cell wall in Ponkan mandarin 1 day after infection with *X. fastidiosa*.** Log_2_ fold change of gene expression (*X. fastidiosa* versus mock inoculated control) was analyzed by MapMan. Blue squares represent upregulated genes, red squares represent downregulated genes and grey circles represent not differentially regulated genes. The color saturation indicates fold change >0.8 and < -0.8. **GSL07** (Ciclev10030560m), glucan synthase-like 7; **CSLA09** (Ciclev10031284m), cellulose synthase like A9; **CESA8** (Ciclev10014155m), cellulose synthase; **CESA4** (Ciclev10018639m), cellulose synthase.

In this study, we observed a significant change in expression of genes involved in secondary metabolism, and cell wall biosynthesis and modification in Ponkan mandarin infected with *X. fastidiosa*. These genes were mapped using MapMan to generate a representative overview (Figure 2 and Additional file 3).

In response to *X. fastidiosa* infection, Ponkan mandarin appears to activate genes for the formation and alteration of secondary xylem cells as a defense mechanism. Induction of the transcription factor AS1, which has a MYB domain (Ciclev10012089m) was also observed. It is suggested that the abundance of MYB proteins in the xylem could be involved in transcriptional regulation of the formation of the secondary xylem [20] (Table 1). Furthermore, a *myo* gene (Ciclev10010780m), which encodes actin, was strongly induced (Table 1). Many studies suggest that actin displays a similar expression pattern to microtubules, which are suggested to determine the location for deposition of the secondary cell wall, cellulose, lignin, hemicellulose, and proteins [21]. Another transcription factor induced in this study was MYB66 (Ciclev10017556m), which is probably involved in the regulation of flavonoids biosynthesis and lignification [22-24] (Table 1). To corroborate these observations, we verified the activation of genes related to phenylpropanoid and flavonoids biosynthesis, which act as anti-pathogenic molecules [25]. Some of them such as phenylalanine ammonium lyase are involved in lignin biosynthesis (Table 1; Figure 2 and Additional file 3). In addition to lignification, callose deposition is also an important defense mechanism in plants and one callose synthase (Ciclev10030560m) was induced in Ponkan mandarin infected with *X. fastidiosa* (Table 1; Figure 2 and Additional file 3). Additionally, genes encoding cellulose synthases (Ciclev10031284m, Ciclev10014155m and Ciclev10018639m) were significantly induced, such as CESA8 and CESA4, which are key enzymes in the biosynthesis of the xylem cell wall [26]. Among the repressed genes it is remarkable the presence of those encoding proteins related to cell wall degradation (Table 1; Figure 2 and Additional file 3). These results indicate that the molecular defense response of Ponkan mandarin against *X. fastidiosa* involves the participation of genes related to cell wall biosynthesis. This could represent an important strategy of the plant for restrict the movement of *X. fastidiosa* through the xylem cells.

### Hormone related-genes in the Ponkan mandarin defense response

**Figure 3:**
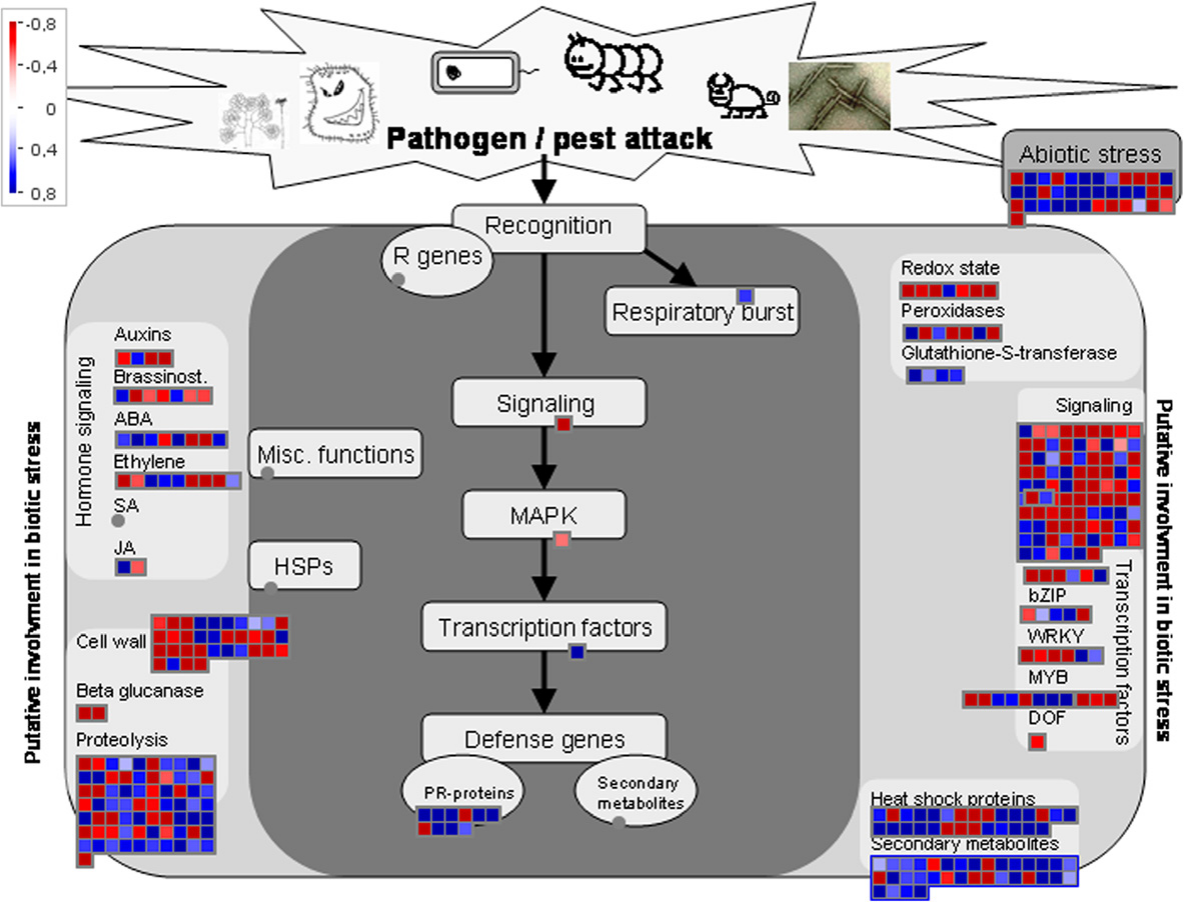
**Stress responses in Ponkan mandarin 1 day after infection with *X. fastidiosa*.** Log_2_ fold change of gene expression (*X. fastidiosa* versus mock-inoculated control) was analyzed by MapMan. Blue squares represent upregulated genes, red squares represent downregulated genes and grey circles represent not differentially regulated genes. The color saturation indicates fold change >0.8 and < -0.8.

After pathogen recognition, plants transmit signals to activate defense responses. This transmission can be performed by secondary messengers, such as G-proteins, Ca^2+^, reactive oxygen species (ROS), nitric oxide, and hormones [27,28]. Genes associated with calcium signaling (Ciclev10000095m, Ciclev10014823m and Ciclev10032932m), G-proteins (Ciclev10014139m, Ciclev10007246m and Ciclev10020496m) and ROS (Ciclev10026073m and Ciclev10026072m) were all upregulated in infected plants (Table 1 and Table 2). In addition, hormone related-genes induced in Ponkan mandarin infected with *X. fastidiosa* were associated with JA, ABA and auxin. JA and ABA pathways are important regulators of expression of defense genes and have been identified downstream in PTI [29,30]. The gene encoding lipoxygenase (LOX), a key enzyme in the synthesis of JA [31], was induced in Ponkan mandarin infected by *X. fastidiosa* (Table 1; Figure 3 and Additional file 4). In addition to the defense response, this hormone activates secondary metabolism in the plant in response to a variety of biotic and abiotic stresses [31,32]. Interestingly, LOX was also upregulated in Ponkan mandarin in later stage of *X. fastidiosa* infection [6,7]. These observations highlight the importance of the JA pathway during the defense response.

**Table 2:** **Defense-genes upregulated in Ponkan mandarin in response to *X. fastidiosa *infection**

**Gene symbol**	Gene id_Citrus clementina*	AGI**	Fold change***	**P ≤ 0.001**	**Gene description**
#	Ciclev10000095m	AT1G20760	0.872938	0.000738646	calcium-binding EF hand family protein
CPK21	Ciclev10014823m	AT4G04720	0.731932	0.0054887	calmodulin-dependent protein kinase
#	Ciclev10032932m	AT1G64850	0.600133	0.0256525	calcium-binding EF hand family protein
#	Ciclev10014139m	AT5G46070	0.882012	0.046128	GTP binding / GTPase
ATMIN7	Ciclev10007246m	AT3G43300	0.625071	0.030625	guanyl-nucleotide exchange factor/ protein binding
#	Ciclev10020496m	AT5G50120	0.617877	0.00757447	WD-40 repeat family protein
PER17	Ciclev10026073m	AT2G22420	0.88525	0.0376681	peroxidase 17
#	Ciclev10026072m	AT4G33420	0.559908	0.00032072	peroxidase, putative
MAP9	Ciclev10021170m	AT1G73500	0.567652	0.000807898	MAP kinase kinase 9
EMB3004	Ciclev10000874m	AT3G06350	1.0645	0.00113401	shikimate dehydrogenase, putative
HSP70	Ciclev10027981m	AT3G12580	1.2447	4.22995e-12	heat shock protein 70
HSP17	Ciclev10009756m	AT5G12020	1.62260	0.00	17.6 kda class II heat shock protein
HSP90	Ciclev10004456m	AT5G52640	1.63013	9.87432e-13	heat shock protein 90.1
DNAJ	Ciclev10028792m	AT4G39150	1.01887	0.00183666	dnaJ heat shock N-terminal domain-containing protein
HPT1	Ciclev10008336m	AT2G18950	2.40936	2.24863e-10	homogentisate phytyltransferase 1
P450	Ciclev10014861m	AT4G12320	1.56043	0.000237455	cytochrome P450, family 706, subfamily A, polypeptide 6
TAU8	Ciclev10005809m	AT3G09270	1.86905	1.54321e-08	glutathione S-transferase TAU 8
UDP-glycosyltransferase	Ciclev10025462m	AT3G50740	1.139	1.72708e-07	udp-glucosyl transferase 72E1
CHIA	Ciclev10005491m	AT5G24090	1.02503	0.00135025	chitinase A
TRYPSIN	Ciclev10022001m	AT1G17860	1.32418	2.00905e-06	trypsin and protease inhibitor family protein

* *Citrus clementina* transcripts identification number (reference genome used for mapping the reads) - http://www.phytozome.org/search.php

** Identification number of the *Arabidopsis thaliana* ortholog of up and down-regulated citrus gene in response to *X. fastidiosa* infection (The Arabidopsis Genome Initiative).

*** Log_2_ fold change values (P ≤ 0.001) obtained from of each infected sample compared to mock-inoculated control.

# unnamed gene symbol.

Upregulated genes in Ponkan mandarin 24 h after infection with *X. fastidiosa* compared with the control.

With regard to ABA-related genes, AP2 (Ciclev10010403m), *nced*6 (Ciclev10006710m), and *ccd*7 (Ciclev10027500m) were induced in response to *X.fastidiosa* infection (Table 1; Figure 3 and Additional file 4). AP2 is involved in the activation of genes related to ABA biosynthesis [33], while *nced*6 and *ccd*7 are associated with biosynthesis and transport of this hormone, respectively. Additionally the *aip*2 gene (Ciclev10008969m) was repressed (Table 1; Figure 3 and Additional file 4) and this gene encodes an E3 ligase that negatively regulates ABA signaling by targeting ABI3, a central regulator of this pathway, for degradation [34].

So the repression of this gene strengthens the idea that ABA biosynthesis is activated in Ponkan mandarin in response to *X. fastidiosa* infection.

**Figure 4:**
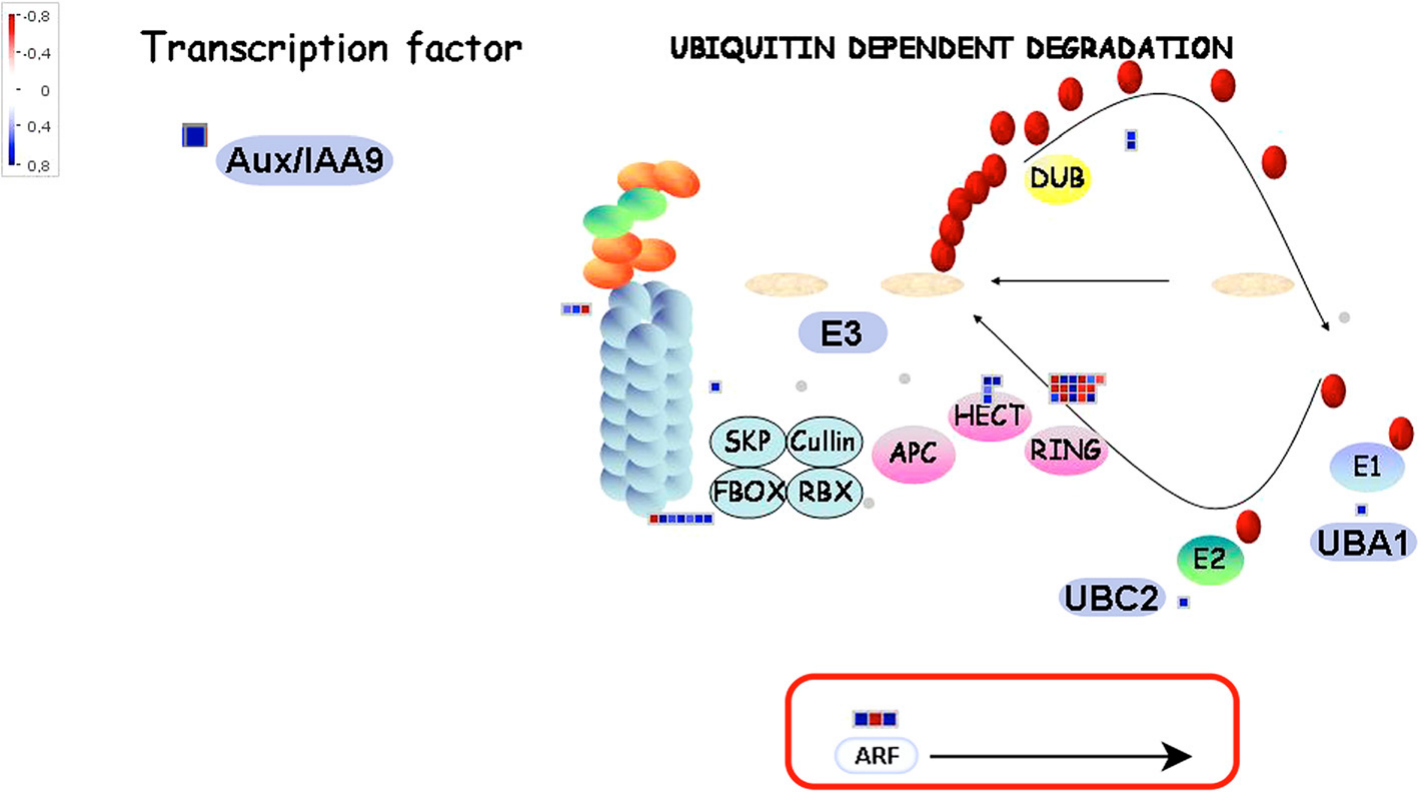
**The ubiquitin dependent degradation system in Ponkan mandarin 1 day after infection with *X. fastidiosa*.** Activation of the auxin pathway in Ponkan mandarin after infection with the bacteria. Signaling of this hormone happens when, at high auxin concentrations, the transcription factor Aux /IAA9 is sent to ubiquitin dependent degradation, which leads to degradation of this factor, allowing the release of auxin responsive factors (ARFs) from the complex. Log_2_ fold change of gene expression (*X. fastidiosa* versus mock-inoculated control) was analyzed by MapMan. Blue squares represent upregulated genes, red squares represent downregulated genes and grey circles represent not differentially regulated genes. The color saturation indicates fold change >0.8 and < -0.8. **Aux /IAA9**, transcription factor Auxin/Indole-3 acetic acid 9; **DUB**, Deubiquitinating enzyme; **E1**, Ubiquitin-activating enzymes; **UBA1**, ubiquitin-activating enzyme E1; **E2**, Ubiquitin-conjugating enzymes; **UBC2**, ubiquitin-conjugating enzyme E2; **RING**, C3HC4 RING-domain-containing ubiquitin E3 ligase; **HECT**, HECT type E3; **APC**, anaphase-promoting complex; **E3**, Ubiquitin ligases; **Cullin**, **SKP**, **FBOX** and **RBX**, subunit of the E3 ligase; **ARF**, auxin responsive factors.

Other hormone related-genes upregulated in Ponkan mandarin in response to infection by *X. fastidiosa* were associated with the auxin signaling pathway (Table 1; Figure 4 and Additional file 5). Indole-3-acetic acid (IAA) is the main auxin in plants, controlling many important physiological processes, including cell growth and division, tissue differentiation and response to light [35,36]. In addition, auxin is also associated with increased susceptibility to biotrophic microorganisms, because it promotes loosening of the cell wall and thus potentiates pathogen growth [30,37]. Many bacteria produce IAA as a strategy to interfere with the plant auxin pathway to facilitate their infection [38]. However, our results showed the induction of several genes involved in the activation of the auxin signaling pathway in a resistant plant after infection, suggesting that the plants do not recognize *X. fastidiosa* as a biotrophic pathogen. The map locations of modulated auxin genes found in this study are shown in representative schematics of auxin synthesis and degradation (Figure 4). At high concentrations, auxin promotes an association between Auxin/IAA (Aux/IAA) and an F-box protein known as transport inhibitor response 1 (TIR1). This complex activates the E3 ligase that leads to degradation of Aux/IAA, allowing the release of auxin responsive factors (ARFs) from the complex [39]. A gene encoding Aux/IAA (IAA9 - Ciclev10025873m) was the most upregulated in our analysis. RING E3 subunit (Ciclev10005027m), a HECT subunit (UPL5 - Ciclev10014213m) and E3/SCF/FBOX (LKP1-Ciclev10007762m) genes, which are all part of the proteasome complex, were also induced. Additionally, an ubiquitin-specific protease (ARF-GAP DOMAIN 8 - Ciclev10011983m) was also significantly induced in the infected plant (Table 1; Figure 4 and Additional file 5). This enzyme is highly conserved in eukaryotes, and plays a critical role by cleaving ubiquitinated proteins [40]. Furthermore, two ARFs (ARF19 and ARF8) related-genes and a gene encoding BIG, which is involved in polar auxin transport and has an essential function in auxin signaling [41] were upregulated (Table 1).

Many studies have reported that auxin promotes susceptibility to bacterial diseases [42,43]. However, this affirmation is only true for biotrophic organisms: auxin signaling is an important component involved in plant resistance to necrotrophic pathogens [30,44]. Auxin signaling is upregulated in Ponkan mandarin in response to *X. fastidiosa* and this plant is resistant to this pathogen; therefore, we speculated that in the early stage of infection Ponkan mandarin recognizes *X. fastidiosa* as a necrotrophic organism, even though this bacterium does not cause massive destruction of host tissue. This is consistent with the direct injection of the bacteria by the insect vector into the xylem vessels, which is composed mainly of dead cells, and the fact that *X. fastidiosa* is able to degrade plant cell wall, which is an important factor for its colonization in susceptible plants [4]. Therefore, substantial tissue destruction is not necessary to trigger necrotroph-related responses mediated by *X. fastidiosa* in the resistant plant host.

The evidence in this paper indicates that activation of the auxin signaling pathway does not promoting susceptibility of Ponkan mandarin after infection with the bacterium. The involvement of this hormone in pathogen susceptibility disease development appears to include rapid elongation of plant tissues by increasing the extensibility of the cell wall [45]. Proteins that participate in acid-induced cell wall extension are endo-β-1,4-glucanases (EGases), xyloglucan endotransglycosylases (XETs), and expansins [46]. We did not observed induction of a major set of these proteins. In fact some of them such as XETs (Ciclev10005561m and Ciclev10028851m) and expansin (Ciclev10012518m) were repressed in Ponkan mandarin in response to *X. fastidiosa*, suggesting that auxin signaling related genes found in this work are not primarily promoting cell expansion (Table 1). On the other hand, auxin synergistically with jasmonic acid/ethylene-signaling is required for necrotrophic resistance. In addition some members of ARF positively regulate camalexin biosynthesis resulting in resistance to necrotrophic pathogens [47]. This defense system, involving the interaction between auxin and JA signaling pathways, which probably occurs in Ponkan mandarin, given the significant induction of genes associated with both signaling pathways. Camalexin is produced through the tryptophan pathway and in our analysis two genes involved in the biosynthesis of tryptophan, dehydroquinate-shikimate dehydrogenase (Ciclev10000874m) and MAPK 9 (Ciclev10021170m), were induced in Ponkan mandarin, suggesting that camalexin biosynthesis may be induced in response to *X. fastidiosa* infection. Taken together all these evidences suggest us that the resistant Ponkan mandarin recognizes *X. fastidiosa* in early stage of infection as a necrotrophic pathogen.

**Figure 5:**
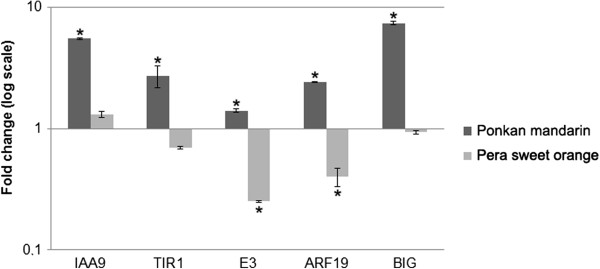
**Relative quantification of genes related auxin pathway in Ponkan mandarin and sweet orange by RT-qPCR.** cDNA samples were prepared using RNA from xylem tissue from Ponkan mandarin and sweet orange, after 1 day of infection with *X. fastidiosa* or not (control) (three biological replicates). The bars indicate the standard deviation of the means. (*) indicates significant difference (P ≥ 0.05) between the mean values obtained for each gene compared with the control.

**Figure 6:**
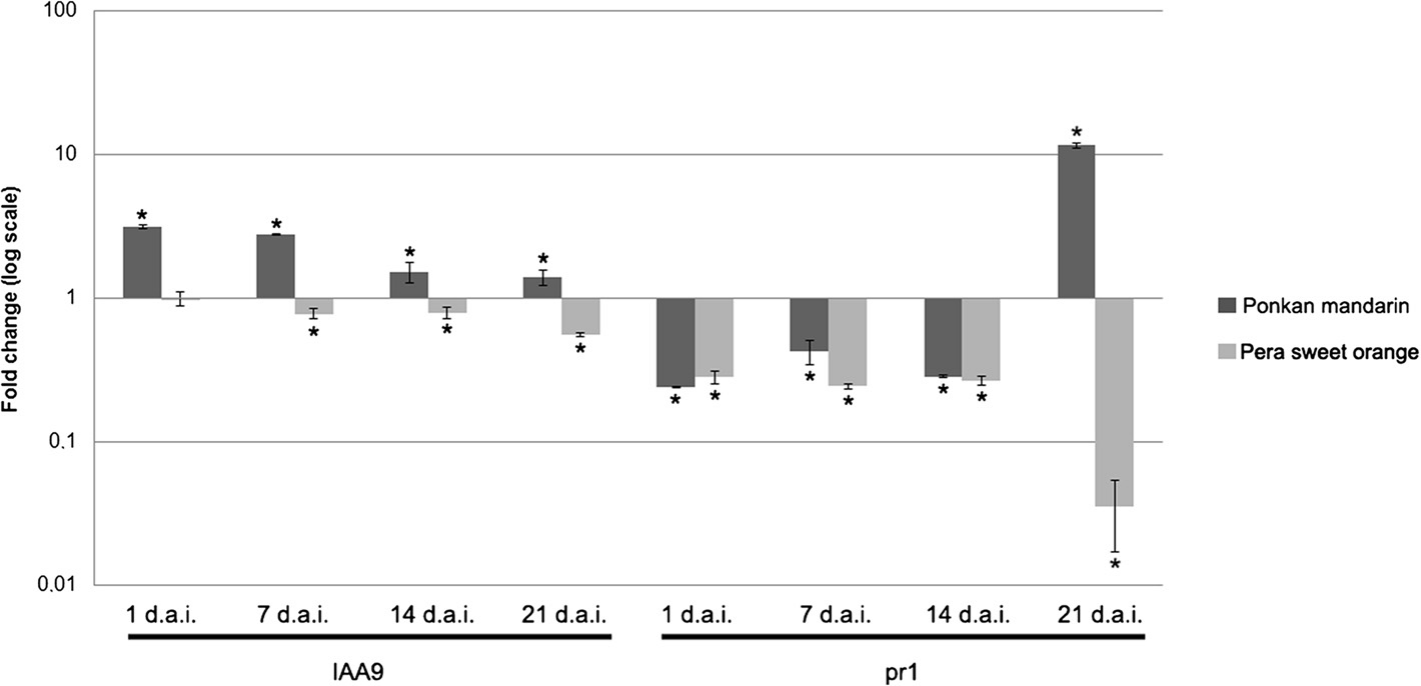
**Relative quantification of genes encoding IAA9 and PR1 in *Citrus *plants infected with *X. fastidiosa *by RT-qPCR.** cDNA samples were prepared using RNA extracted from a mixture of leaves and petioles of Ponkan mandarin and Pera sweet orange at 1, 7, 14, and 21 days after inoculation (d.a.i.) of *X. fastidiosa* or mock inoculated (control). The experiment was conducted with three biological replicates. The bars indicate the standard deviations of the means. (*) indicates significant difference (P ≥ 0.05) between the mean values obtained for each gene compared to control.

To confirm that auxin signaling related genes are indeed upregulated only in Ponkan mandarin in response to *X. fastidiosa* infection we also evaluated the expression of auxin marker-genes by RT-qPCR in Pera sweet orange susceptible variety and in Ponkan mandarin at one day after *X. fastidiosa* infection. As shown in Figure 5, all auxin related-genes were significantly induced only in Ponkan mandarin. In Pera sweet orange, the genes were significantly repressed (E3 and ARF19) or showed no significant change (IAA9, TIR1 and BIG). This result evidences that auxin is induced as a resistance response against *X. fastidiosa* during the early stage of infection. Recognition of PAMPs or DAMPs that somehow resemble necrotrophic pathogens may mediate this response. However, this recognition occurs mainly during the early stage of infection since we observed a gradual decrease in expression of auxin related-genes along the time course of infection (Figure 6). After 21 days, no auxin related-gene was expressed, whereas expression of salicylic acid (SA) marker-gene increased (Figure 6). This result agrees with De Souza et al. [6,7] where an upregulation of SA related-genes was observed in Ponkan mandarin at 30 days after *X. fastidiosa* inoculation. After this time point, the bacterial population decreases to a point where it could not be isolated [3]. These results suggest that the resistant plant changes its mechanism of defense during *X. fastidiosa* infection: the initial response involves the participation of auxin while later on SA becomes important. It is to note that the change occurs approximately at the time when *X. fastidiosa* forms a structured biofilm. In this growth condition this bacterium expresses specific genes and proteins necessary for its adaptation and pathogenicity in the host [3,4,48,49]. Therefore other proteins expressed in biofilm condition could be later recognized by the plant. Nevertheless, how the resistant plant indeed recognizes *X. fastidiosa* to trigger different pathways in the resistance response remains to be discovered. Other downstream defense-genes upregulated in Ponkan mandarin after *X. fastidiosa* infection are represented at the Table 2. These genes might contribute to increase the resistance response in Ponkan mandarin to *X. fastidiosa* culminating in its elimination in the plant.

To confirm the participation of different genes related to pathogen recognition, cell wall synthesis, and hormone signaling pathways in the mandarin resistance response, we also tested their expression in Pera sweet orange. The analysis confirmed that genes encoding LRR-RLK and CC-NBS-LRR (pathogen recognition), AP2 (ABA signaling), MYO and CESA4 (cell wall synthesis) were not only specifically induced in mandarin but also repressed in sweet orange (Additional file 6).

### Validation of RNA-seq data by RT-qPCR

**Figure 7:**
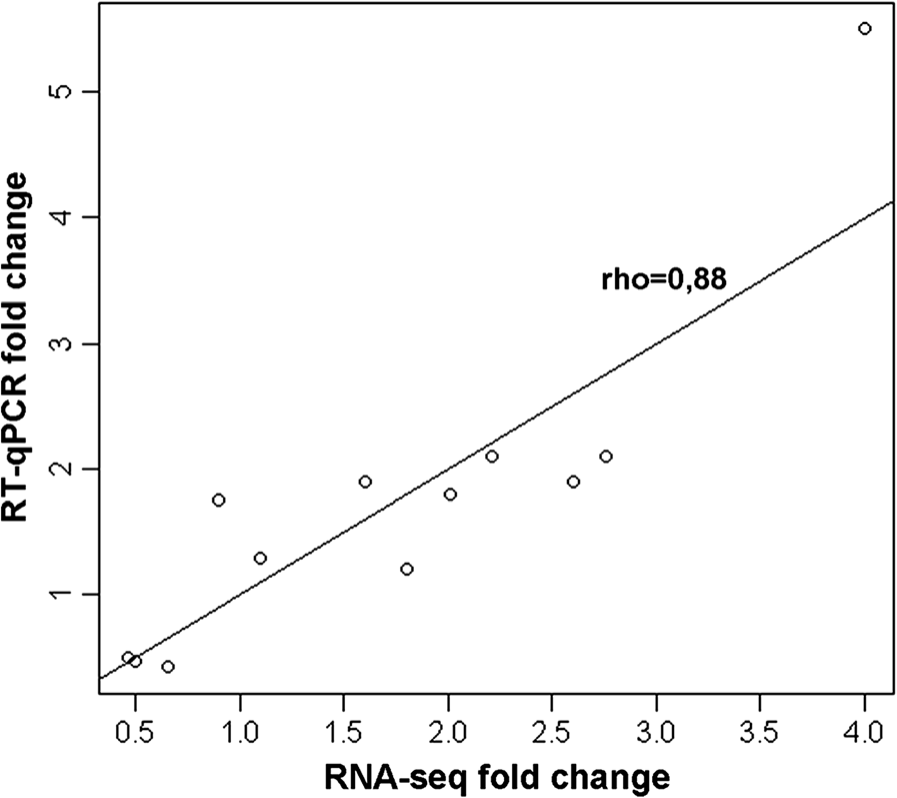
**Correlation between RNA-seq and RT-qPCR data.** Twelve differentially expressed genes in Ponkan mandarin challenged with *X. fastidiosa* were selected for validation.

RT-qPCR was used to validate the RNA-seq data. Twelve genes involved in different biological processes were selected (Additional file 7). Similar expression patterns were observed for all genes evaluated by both techniques (Additional file 8). Additionally, a high Spearman’s rho value (0.88) indicated a good correlation between the fold change from RNA-seq experiments and RT-qPCR. These results confirmed the reliability and accuracy of the RNA-Seq data in this study (Figure 7).

## Conclusions

This work demonstrated that the defense response of Ponkan mandarin to *X. fastidiosa* involves induction of genes related to PAMP receptors, cell wall synthesis, and ABA, JA and auxin signaling pathways, which will probably culminate in the induction of other defense-related genes. We also hypothesize that Ponkan mandarin initially recognizes *X. fastidiosa* as a necrotrophic pathogen, inducing genes related to the auxin signaling pathway and later it changes the response to a biotrophic pathogen.

The genes found in this work are important tools to be used in breeding programs to accelerate the selection of hybrid from *C. reticulata* and *C. sinensis* carrying defense responses from mandarin. Moreover these genes can be also used for genetic engineering aiming the development of resistant sweet orange varieties.

## Methods

### Plant materials and bacterial detection

The experiments were conducted with physiologically mature plants, of uniform size and production of young leaves. When the shoots reached a length of approximately 10 cm, they were artificially inoculated by needle prick with 10 μL suspension (10^8^ cells mL^-1^) of *X. fastidiosa* strain 9a5c in PBS buffer at five different points on the same stem. Negative controls comprising Ponkan mandarin (*C. reticulata* Blanco) and Pera sweet orange (*Citrus sinensis* L. Osb) were mock inoculated with PBS buffer.

Total genomic DNA (plant + bacteria) was extracted from inoculated cambial tissue enriched with xylem after 1 day using a CTAB method adapted from [50]. These samples were used for detection of bacteria using real-time PCR reactions. The analyses were carried out with an ABI PRISM 7500 Sequence Detector System (Applied Biosystems, Foster City, CA, USA). The reaction was performed in a total volume of 25 μL, containing 12.5 μL of TaqMan PCR Master Mix fast (Applied Biosystems), 200 ng of DNA template and 525 nM of primers CVC-1 and CCSM-1 [51]. Each sample was tested in triplicate and with five biological replicates. Negative (no template DNA) and positive (DNA from *X. fastidiosa*) controls were included in all experiments to exclude or detect any possible contamination. The samples were considered positive for the presence of *X. fastidiosa* when they presented Ct (Cycle Threshold) below or equal to 37; negative samples did not present amplification up to this Ct value (unpublished data). From these results, we selected the three biological replicates for different conditions and performed transcriptome analyses.

### RNA isolation and expression analysis in RNA-seq

For transcriptome analysis we used cambial tissue enriched with xylem from Ponkan mandarin. Total RNA was extracted with Trizol (Invitrogen Life Technologies Foster City, CA, USA) and treated with DNase RNase-Free Set (Qiagen, Valencia, CA, USA), according to the manufacturer’s instructions. We extracted RNA samples from a pool of three independent biological replicates infected after one day and their respective controls (not infected). The concentration of RNA was measured in a NanoDrop ND-1000 spectrophotometer (NanoDrop Technologies, Wilmington, DE). RNA quality was evaluated using an Agilent Bioanalyzer Model 2100 (Agilent Technologies, Palo Alto, CA).

A total of 10 μg of RNA from Ponkan mandarin (control and infected with *X. fastidiosa*) were sent to Macrogen Inc. (South Korea) for sequencing using the Genome Analyzer IIx platform (Illumina Inc.). All procedures were performed according to Illumina’s protocols. Purified cDNA libraries were dispersed onto an Illumina single-end flow cell composed of eight lanes using the Illumina Cluster Station (Illumina Inc.). One lane was used per sample of the treated and control plants. The 101 bp reads were collected using the Illumina GA II and sequencing-by-synthesis technology. The sequences of Ponkan mandarin were mapped against the *Citrus clementina* reference genome (http://www.phytozome.net/clementine.php) using TopHat [52]. After alignment, the relative abundance of the transcripts was measured with the Cufflink software, which measures the transcripts abundance as RPKM (Reads Per Kilobase of exon model per Million mapped reads). The differential expression between Ponkan mandarins inoculated or not with bacteria, and its significance, was calculated in Cuffdiff [53]. The differentially expressed transcripts were annotated and automatically categorized using GO (Gene Ontology - http://www.blast2go.com/b2ghome). These sequences were also used to search for similar protein sequences available in GenBank using the BLASTX tool.

In addition, the differentially expressed genes were also functionally analyzed using the MapMan software, which is a user-driven tool that displays large genomics datasets onto diagrams of metabolic pathways or other processes [54].

### Expression analysis by RT-qPCR

Twelve genes that were identified by RNA-seq to be induced or repressed in Ponkan mandarin in response to *X. fastidiosa*, were selected for validation by RT-qPCR: *ATEXPA*4; *CLV*1; *CC-NBS-LRR*; *RLK*; *P*12; *LOX*; *AIP*; *MYO*; *AP*2; *HSP*90; *CCR*4 and *IAA*9 (Additional file 7).

Furthermore, we used RT-qPCR to compare the level of expression of some genes involved in the auxin pathway, pathogen recognition, ABA signal transduction, and cell wall synthesis in Ponkan mandarin and in Pera sweet orange, a susceptible variety, one day after infection with the bacteria. We evaluated the *IAA9* (Aux/IAA), *ARF*19, *TIR*1, *BIG* and *E*3 genes (auxin pathway); LRR-RLK and CC-NBS-LRR (pathogen recognition); AP2 (ABA); MYO and CESA4 (cell wall synthesis).

Additionally, we checked the relative quantification of genes encoding IAA9 and PR1 in *Citrus* plants, by RT-qPCR, using RNA extracted from a mixture of leaves and petioles of Ponkan mandarin and Pera sweet orange at 1, 7, 14, and 21 days after inoculation of *X. fastidiosa* or mock inoculated (control).

The primers for these genes were designed using PrimerExpress software (Applied Biosystems, Foster City, CA, USA) (Additional file 9).

The specificity of the primers was checked in silico against the NCBI database (http://www.ncbi.nlm.nih.gov/) using the Primer-BLAST tool. All the primer sequences showed specificity with the sequences of target genes. Additionally, we checked the pattern of dissociation obtained after RT-qPCR, using a meting curve for each primer. This showed a single peak for all the evaluated genes, confirming the existence of only one amplicon (Additional file 10).

The efficiency of the primers was estimated in each experiment using the software Miner (http://www.miner.ewindup.info/). This software quantifies the results of RT-qPCR based on the kinetics of the PCR amplification individually for each sample, without the need for a standard curve. This allows a direct calculation of the efficiency and values of cycle quantification (cq) [55]. All primers showed amplification efficiencies between 90 - 100% (Additional file 10).

To find a reference gene to normalize the RT-qPCR results, the stability of five endogenous control genes in *Citrus* was analyzed to confirm their stability using geNorm software [56] and to ensure the existence of gene expression variation due to the experimental conditions. The primers for these genes were obtained from a previous work [57]. In this evaluation we used samples of Pera sweet orange and Ponkan mandarin (control and infected with *X. fastidiosa*). Ubiquitin (UBQ) and cyclophilin (CYC) were the most stable and were selected for further analysis. However, the other three genes, eukaryotic translation elongation factor 2, NADP-isocitrate dehydrogenase and tubulin also showed satisfactory mean values (*M-value*) (Additional file 11). These *M-values* are within acceptable values at a cutoff value of 0.15 [57].

For the analyses of gene expression by RT-qPCR, we used RNA isolated as described above, with three independent biological replicates, infected or not with *X. fastidiosa*. These RNAs were used for the cDNA synthesis according to the instructions of the Thermo Scientific for the RevertAid H Minus First Strand cDNA Synthesis Kit. After synthesis, the cDNAs were diluted at 1:25 and used in RT-qPCR. The evaluations were performed on an ABI Prism 7500 Sequence Detector System (Applied Biosystems, Foster City, CA, USA) using absolute quantification analysis. The detection of PCR products was measured by monitoring the increase in fluorescence emitted by SYBR green marker. For all amplifications performed in RT-qPCR, we produced dissociation curves to check for nonspecific amplification resulting from possible contamination.

The analysis and normalization of gene expression were performed in the Genex software (version 5.0.1.5; http://www.multid.se), using the efficiencies and Cqs generated in the Miner software, and transformed into non-standardized data (Q). Uninfected samples were used as calibrators for each genotype evaluated. We used two endogenous genes for the normalization of the data.

## Competing interests

The authors declare that they have no competing interests.

## Authors’ contributions

MAM and AAS planned and supervised the study. CMR and AAS contributed to the design and execution of the experiments. CMR conducted inoculation, collection, processing of samples, functional analysis of differentially expressed genes and drafted the manuscript. LTK and MAT contributed to the RNA-seq analysis, categorization and annotations of differentially expressed transcripts. CMR, AAS, MAT and MAM contributed to the interpretation of the data and provided intellectual input. MAT, AAS, LTK and MAM revised the manuscript. All authors read and approved the final manuscript.

## Supplementary Material

Additional file 1 **Detection of *X. fastidiosa* in plants of Ponkan mandarin and Pera sweet orange by RT-qPCR.** DNA samples were prepared from xylem tissue from Ponkan mandarin and Pera sweet orange, after infection with *X. fastidiosa* or not (control) for one day, with five biological replicates for each species and their respective controls. (1-5c) Pera sweet orange control; (6-10c) Ponkan mandarin control; (1-5d) Pera sweet orange with *X. fastidiosa*; (6-10d) Ponkan mandarin infected with *X. fastidiosa*; (c +) Positive control of a plant with CVC. Y-axis represents cycle quantification (Cq) as determined by RT-qPCR. Click here for file

Additional file 2 **Differentially expressed genes in Ponkan mandarin after infection by *X. fastidiosa* (P ≤ 0.001).** Up and downregulated genes in Ponkan mandarin 24 h after infection with *X. fastidiosa* compared with the control. Click here for file

Additional file 3 **Differentially expressed genes involved in the cell wall and secondary metabolism, according to MapMan results.** Up and downregulated genes in Ponkan mandarin 24 h after infection with *X. fastidiosa* compared with the control that are related to secondary metabolism and the cell wall. Click here for file

Additional file 4 **Differentially expressed genes involved in biotic stress, according to MapMan results.** Up and downregulated genes related to biotic stress in tangerine Ponkan mandarin 24 h after infection with *X. fastidiosa*. Click here for file

Additional file 5 **Differentially expressed genes involved in systemic ubiquitin dependent degradation, according to MapMan results.** Up and downregulated genes related to ubiquitin dependent degradation probably involved in auxin pathway in Ponkan mandarin 24 h after infection with *X. fastidiosa*. Click here for file

Additional file 6 **Relative quantification of genes related to pathogen recognition, cell wall synthesis, and hormone signaling pathways in *Citrus* plants infected with *X. fastidiosa* by RT-qPCR.** cDNA samples were prepared using RNA from xylem tissue from Ponkan mandarin and Pera sweet orange, after 1 day of infection with or without (control) *X. fastidiosa* (three biological replicates). The bars indicate the standard deviation of the means. (*) indicates significant difference (P ≥ 0.05) between the mean values obtained for each gene [LRR-RLK and CC-NBS-LRR (pathogen recognition); AP2 (ABA); MYO and CESA4 (cell wall synthesis)] compared with the control. Click here for file

Additional file 7 **Genes involved in different biological processes selected from RNA-seq Ponkan mandarin infected with *X. fastidiosa*.** Twelve differentially expressed genes in Ponkan mandarin infected with *X. fastidiosa* were selected to validate the results obtained from RNA-seq analysis. Click here for file

Additional file 8 **Validation of 12 differentially expressed genes selected from RNA-seq analysis by RT-qPCR.** The fold changes are shown for 12 differentially expressed genes identified using RNA-seq compared to those obtained by RT-qPCR. For this, cDNAs were prepared from RNA of Ponkan mandarin xylem tissue infected with *X. fastidiosa* or not (control) after one day, with three biological replicates. RT-qPCR data were normalized to the two most stable endogenous control genes (UBQ and CYP). Click here for file

Additional file 9 **Genes and primers used in RT-qPCR.** Includes sequences of oligonucleotide primers used for RT-qPCR analysis. Click here for file

Additional file 10 **Validation of the specificity and amplification efficiency of the RT-qPCR primers.** Amplification of cDNA for Ponkan mandarin genes *ATEXPA*4, *CLV*1, *CC-NBS-LRR*, *LRR-RLK, P12*, *LOX*, *AIP*, *MYO*, *AP*2, *HSP*90, *CCR*4, *IAA*9, *ARF*19, *TIR*1, *BIG, E*3, *PR*1 and *CESA*4. (A) Verification the dissociation pattern obtained after RT-qPCR. Each gene showed a single peak after melting curve analysis, confirming the specificity the primers. (B) Efficiency of amplification using primers obtained through Miner software after RT-qPCR. Click here for file

Additional file 11 **Average expression stability values (M) of the five citrus endogenous control genes calculated by geNorm.** Expression stability values were calculated for samples from citrus genotypes infected with *X. fastidiosa* and controls (mock). A lower M value indicates more stable expression. Click here for file

## References

[B1] Bovè JM Ayres AJ Etiology of three recent diseases of citrus in São Paulo state: sudden death, Variegated Chlorosis and Huanglongbing IUBMB Life 2007 59 4–5 346 354 17505974 10.1080/15216540701299326

[B2] Hopkins DL *Xylella fastidiosa* xylem-limited bacterial pathogen of plants Annu Rev Phytopathol 1989 27 271 290 10.1146/annurev.py.27.090189.001415

[B3] De Souza AA Takita MA Coletta-Filho HD Caldana C Goldman GH Yanai GM Muto NH Oliveira RC Nunes LR Machado MA Analysis of gene expression in two growth states of *Xylella fastidiosa* and its relationship with pathogenicity Mol Plant Microbe Interact 2003 16 867 875 10.1094/MPMI.2003.16.10.867 14558688

[B4] Pérez-Donoso AG Sun Q Roper MC Greve LC Kirkpatrick B Labavitch JM Cell wall-degrading enzymes enlarge the pore size of intervessel pit membranes in healthy and *Xylella fastidiosa*-infected grapevines Plant physiol 2010 152 3 1748 1759 10.1104/pp.109.148791 20107028 PMC2832268

[B5] Coletta-Filho HD Pereira EO De Souza AA Takita MA Cristofani-Yaly M Machado MA Analysis of the resistance to *Xylella fastidiosa* within a hybrid population of Pera sweet orange and Murcott tangor Plant Pathol 2007 56 661 668 10.1111/j.1365-3059.2007.01605.x

[B6] De Souza AA Takita MA Coletta-Filho HD Campos MA Teixeira JEC Targon MLPN Carlos EF Ravasi JR Fischer CN Machado MA Comparative analysis of differentially expressed sequence tags of sweet orange and mandarin infected with *Xylella fastidiosa* Genet Mol Biol 2007 30 3 965 971 10.1590/S1415-47572007000500024

[B7] De Souza AA Takita MA Amaral AM Coletta-Filho HD Machado MA Citrus responses to *Xylella fastidiosa* infection, the causal agent de citrus variegated chlorosis Tree For Sci Biotech 2009 2 3 957 964

[B8] Jones JDG Dangl JL The plant immune system Nature 2006 444 323 329 10.1038/nature05286 17108957

[B9] Nicaise V Roux M Zipfel C Recent advances in PAMP-triggered immunity against bacteria: pattern recognition receptors watch over and raise the alarm Plant Physiol 2009 150 1638 1647 10.1104/pp.109.139709 19561123 PMC2719144

[B10] Lehti-Shiu MD Zou C Hanada K Shiu S-H Evolutionary history and stress regulation of plant receptor-like Kinase/Pelle genes Plant Physiol 2009 150 12 26 10.1104/pp.108.134353 19321712 PMC2675737

[B11] Boller T He SY Innate immunity in plants: an arms race between pattern recognition receptors in plants and effectors in microbial pathogens Science 2009 324 5928 742 744 10.1126/science.1171647 19423812 PMC2729760

[B12] Mizuno S Osakabe Y Maruyama K Ito T Osakabe K Sato T Shinozaki K Yamaguchi-Shinozaki K Receptor-like protein kinase 2 (RPK 2) is a novel factor controlling anther development in *Arabidopsis thaliana* Plant J 2007 50 5 751 766 10.1111/j.1365-313X.2007.03083.x 17419837

[B13] Bleckmann A Weidtkamp-Peters S Seidel CAM Simon RD Stem cell signaling in Arabidopsis requires CRN to localize CLV2 to the plasma membrane Plant Physiol 2010 152 166 176 10.1104/pp.109.149930 19933383 PMC2799354

[B14] Wang G Long Y Thomma BPHJ De Wit PJGM Angenent GC Fiers M Functional analyses of the CLAVATA2-like proteins and their domains that contribute to CLAVATA2 specificity Plant Physiol 2010 152 320 331 10.1104/pp.109.148197 19897604 PMC2799366

[B15] Simpson AJG Reinach FC Arruda P The genome sequence of the plant pathogen *Xylella fastidiosa* Nature 2000 40 151 159 10910347 10.1038/35018003

[B16] Abramovitch RB Anderson JC Martin GB Bacterial elicitation and evasion of plant innate immunity Nat Rev Mol Cell Biol 2006 7 8 601 611 10.1038/nrm1984 16936700 PMC2842591

[B17] Lotze MT Zeh HJ Rubartelli A Sparvero LJ Amoscato AA Washburn NR DeVera ME Liang X Tör M Billiar T The grateful dead: damage associated molecular pattern molecules and reduction/oxidation regulate immunity Immunol Rev 2007 220 60 81 10.1111/j.1600-065X.2007.00579.x 17979840

[B18] Boller T Georg F A renaissance of elicitors: perception of microbe-associated molecular patterns and danger signals by pattern-recognition receptors Annu Rev Plant Biol 2009 60 379 406 10.1146/annurev.arplant.57.032905.105346 19400727

[B19] Salomon D Sessa G Identification of growth inhibition phenotypes induced by expression of bacterial type III effectors in yeast J Vis Exp 2012 37 e1865 10.3791/1865 PMC3168205 20354502

[B20] Newman LJ Campbell MM Savidge RA, Barnett JR, Napier R MYB proteins and xylem differentiation Cell and molecular biology of wood formation 1999 Oxford, UK: Bios Academic Publishing 437 444

[B21] Hogetsu T Mechanism for formation of the secondary wall thickening in tracheary elements-microtubules and microfibrils of tracheary elements of Pisum-Sativum L and Commelina-Communis L and the effects of Amiprophosmethyl Planta 1991 185 190 200 24186341 10.1007/BF00194060

[B22] Tamagnone L Merida A Parr A Mackay S Culianez-Macia FA The AmMYB308 and AmMYB330 transcription factors from antirrhinum regulate phenylpropanoid and lignin biosynthesis in transgenic tobacco Plant Cell 1998 10 135 154 9490739 10.1105/tpc.10.2.135 PMC143979

[B23] Patzlaff A McInnis S Courtenay A Surman C Newman LJ Characterization of a pine MYB that regulates lignification Plant J 2003 36 743 754 10.1046/j.1365-313X.2003.01916.x 14675440

[B24] Fornale S Shi X Chai C Encina A Irar S ZmMYB31 directly represses maize lignin genes and redirects the phenylpropanoid metabolic flux Plant J 2010 64 633 644 10.1111/j.1365-313X.2010.04363.x 21070416

[B25] De Luca V St Pierre B The cell and developmental biology of alkaloid biosynthesis Trends Plant Sci 2000 5 4 168 173 10.1016/S1360-1385(00)01575-2 10740298

[B26] Taylor NG Howells RM Huttly AK Vickers K Turner SR Interactions among three distinct CesA proteins essential for cellulose synthesis Proc Natl Acad Sci USA 2003 100 3 1450 1455 10.1073/pnas.0337628100 12538856 PMC298793

[B27] Nürnberger T Scheel D Signal transmission in the plant immune response Trends Plant Sci 2001 6 8 372 379 10.1016/S1360-1385(01)02019-2 11495791

[B28] Lecourieux D Ranjeva R Pugin A Calcium in plant defence-signalling pathways New Phytol 2006 171 2 249 269 10.1111/j.1469-8137.2006.01777.x 16866934

[B29] Melotto M Underwood W Koczan J Nomura K He SY Plant Stomata Function in Innate Immunity against Bacterial Invasion Cell 2006 126 969 980 10.1016/j.cell.2006.06.054 16959575

[B30] Bari R Jones JDG Role of plant hormones in plant defence responses Plant Mol Biol 2009 69 473 488 10.1007/s11103-008-9435-0 19083153

[B31] Delker C Stenzel I Hause B Miersch O Feussner I Wasternack C Jasmonate biosynthesis in *Arabidopsis thaliana*-enzymes, products, regulation Plant Biol 2006 8 3 297 306 10.1055/s-2006-923935 16807821

[B32] Clarke SM Cristescu SM Miersch O Harren FJ Wasternack C Mur LA JAs act with salicylic acid to confer basal thermotolerance in *Arabidopsis thaliana* New Phytol 2009 182 175 187 10.1111/j.1469-8137.2008.02735.x 19140948

[B33] Zhou J Zhang H Yang Y Zhang Z Zhang H Abscisic acid regulates TSRF1-mediated resistance to *Ralstonia solanacearum* by modifying the expression of GCC box-containing genes in tobacco J Exp Bot 2008 59 3 645 652 10.1093/jxb/erm353 18252700

[B34] Zhang X Garreton V Chua N-H The AIP2 E3 ligase acts as a novel negative regulator of ABA signaling by promoting ABI3 degradation Gene Dev 2005 19 1532 1543 10.1101/gad.1318705 15998807 PMC1172060

[B35] Woodward AW Bartel B Auxin: regulation, action, and interaction Ann Bot 2005 95 707 735 10.1093/aob/mci083 15749753 PMC4246732

[B36] Paponov IA Teale WD Trebar M Blilou K Palme K The PIN auxin efflux facilitators: evolutionary and functional perspectives Trends Plant Sci 2005 10 170 177 10.1016/j.tplants.2005.02.009 15817418

[B37] Ding X Cao Y Huang L Zhao J Xu C Li X Wang S Activation of the indole-3-acetic acid-amidosynthetase GH3-8 suppresses expansin expression and promotes salicylate- and jasmonate-independent basal immunity in rice Plant Cell 2008 20 228 240 10.1105/tpc.107.055657 18192436 PMC2254934

[B38] Reineke G Heinze B Schirawski J Buettner H Kahmann R Basse CW Indole-3-acetic acid (IAA) biosynthesis in the smut fungus Ustilagomaydis and its relevance for increased IAA levels in infected tissue and host tumour formation Mol Plant Pathol 2008 9 339 355 10.1111/j.1364-3703.2008.00470.x 18705875 PMC6640242

[B39] Kazan K Manners JM Linking development to defense: auxin in plant–pathogen interactions Trends Plant Sci 2009 14 7 373 382 10.1016/j.tplants.2009.04.005 19559643

[B40] Liu Y Wang F Zhang H He H Ma L Deng XW Functional characterization of the *Arabidopsis* ubiquitin-specific protease gene family reveals specific role and redundancy of individual members in development Plant J 2008 55 844 856 10.1111/j.1365-313X.2008.03557.x 18485060

[B41] Gil P Dewey E Friml J Zhao Y Snowden KC BIG: a calossin-like protein required for polar auxin transport in *Arabidopsis* Gene Dev 2001 15 15 1985 1997 10.1101/gad.905201 11485992 PMC312751

[B42] Navarro L Dunoyer P Jay F Arnold B Dharmasiri N A plant miRNA contributes to antibacterial resistance by repressing auxin signaling Science 2006 312 436 439 10.1126/science.1126088 16627744

[B43] Wang D Pajerowska-Mukhtar K Culler AH Dong X Salicylic acid inhibits pathogen growth in plants through repression of the auxin signaling pathway Curr Biol 2007 17 1784 1790 10.1016/j.cub.2007.09.025 17919906

[B44] Llorente F Muskett P Sánchez-Vallet A López G Ramos B Repression of the auxin response pathway increases *Arabidopsis* susceptibility to necrotrophic fungi Mol Plant 2008 1 496 509 10.1093/mp/ssn025 19825556

[B45] Cosgrove DJ How do plant cell walls extend? Plant Physiol 1993 102 1 1 6 11536544 10.1104/pp.102.1.1 PMC158739

[B46] Catalá C Rose JKC Bennett AB Auxin regulation and spatial localization of an endo-1,4-b-d-glucanase and a xyloglucan endotransglycosylase in expanding tomato hypocotyls Plant J 1997 12 417 426 10.1046/j.1365-313X.1997.12020417.x 9301092

[B47] Fu J Wand S Insights into auxin signaling in plant–pathogen interactions Plant Sci 2011 2 74 1 7 10.3389/fpls.2011.00074 PMC3355572 22639609

[B48] De Souza AA Takita MA Coletta-Filho HD Caldana C Yanai GM Muto NH Costa De Oliveira R Nunes LR Machado MA Gene expression profile of the plant pathogen *Xylella fastidiosa* during biofilm formation in vitro FEMS Microbiol Lett 2004 237 41 353 10.1111/j.1574-6968.2004.tb09676.x 15321682

[B49] De Souza AA Takita MA Pereira EO Coletta-Filho HD Machado MA Expression of pathogenicity-related genes of *Xylella fastidiosa* in vitro and in plant Curr Microbiol 2005 50 223 228 10.1007/s00284-004-4447-8 15902471

[B50] Murray MG Thompson WF Rapid isolation of high molecular weight plant DNA Nuclec Acids Res 1980 8 4321 4325 10.1093/nar/8.19.4321 PMC324241 7433111

[B51] Oliveira AC Garcia AN Cristofani M Machado MA Identification of citrus hybridis through the combination of leaf apex morphology and SSR markers Euphytic 2002 128 397 403 10.1023/A:1021223309212

[B52] Trapnell C Pachter L Salzberg SL TopHat: discovering splice junctions with RNA-Seq Bioinformatics 2009 25 9 1105 1111 10.1093/bioinformatics/btp120 19289445 PMC2672628

[B53] Trapnell C Williams BA Pertea G Mortazavi A Kwan G Van Baren MJ Salzberg SL Wold BJ Pachter L Transcript assembly and quantification by RNASeq reveals unannotated transcripts and isoform switching during cell differentiation Nat Biotechnol 2010 28 5 511 518 10.1038/nbt.1621 20436464 PMC3146043

[B54] Thimm O Bläsing O Gibon Y Nagel A Meyer S Krüger P Selbig J Müller LA Rhee SY Stitt M MAPMAN: a user-driven tool to display genomics data sets onto diagrams of metabolic pathways and other biological processes Plant J 2004 37 6 914 939 10.1111/j.1365-313X.2004.02016.x 14996223

[B55] Artico S Nardeli SM Brilhante O Grossi-De-Sa MF Alves-Ferreira M Identification and evaluation of new reference genes in *Gossypium hirsutum* for accurate normalization of real-time quantitative RT-PCR data BMC Plant Biol 2010 21 10 49 10.1186/1471-2229-10-49 PMC2923523 20302670

[B56] Vandesompele J De Pretter K Pattyn F Poppe B Roy NV De Paepe A Speleman F Accurate normalization of real-time RT-PCR data by geometric averaging of multiple internal control genes Genome Biol 2002 3 7 1 12 10.1186/gb-2002-3-7-research0034 PMC126239 12184808

[B57] Boava LP Laia ML Jacob TR Dabbas KM Gonçalves JF Ferro JA Ferro MIT Furtado EL Selection of endogenous genes for gene expression studies in Eucalyptus under biotic (*Puccinia psidii*) and abiotic (acibenzolar-S-methyl) stresses using RT-qPCR BMC Res Notes 2010 43 3 1 9 10.1186/1756-0500-3-43 PMC2854107 20181283

