# Supplementary figures and images for: RNA-Seq analysis of Citrus reticulata in the early stages of Xylella fastidiosa infection reveals auxin-related genes as a defense response
Source: BMC Genomics. 2013 Oct 3;14:676. doi: 10.1186/1471-2164-14-676 (PMC3852278; doi:10.1186/1471-2164-14-676)

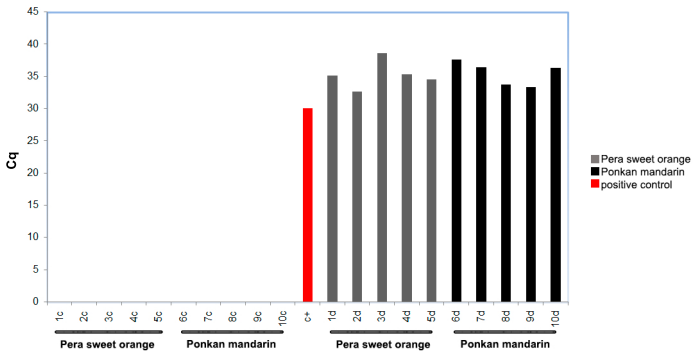

Supplement: Additional file 1 — Detection of X. fastidiosa in plants of Ponkan mandarin and Pera sweet orange by RT-qPCR. DNA samples were prepared from xylem tissue from Ponkan mandarin and Pera sweet orange, after infection with X. fastidiosa or not (control) for one day, with five biological replicates for each species and their respective controls. (1-5c) Pera sweet orange control; (6-10c) Ponkan mandarin control; (1-5d) Pera sweet orange with X. fastidiosa; (6-10d) Ponkan mandarin infected with X. fastidiosa; (c +) Positive control of a plant with CVC. Y-axis represents cycle quantification (Cq) as determined by RT-qPCR. [file 1471-2164-14-676-S1.pdf]

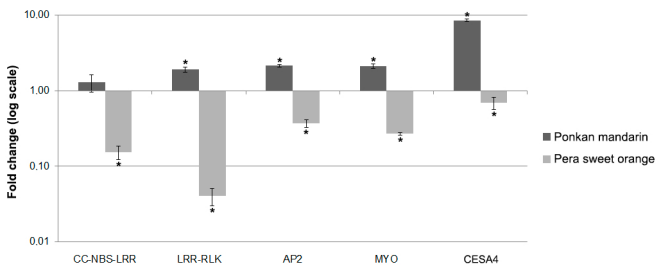

Supplement: Additional file 6 — Relative quantification of genes related to pathogen recognition, cell wall synthesis, and hormone signaling pathways in Citrus plants infected with X. fastidiosa by RT-qPCR. cDNA samples were prepared using RNA from xylem tissue from Ponkan mandarin and Pera sweet orange, after 1 day of infection with or without (control) X. fastidiosa (three biological replicates). The bars indicate the standard deviation of the means. (*) indicates significant difference (P ≥ 0.05) between the mean values obtained for each gene [LRR-RLK and CC-NBS-LRR (pathogen recognition); AP2 (ABA); MYO and CESA4 (cell wall synthesis)] compared with the control. [file 1471-2164-14-676-S6.pdf]

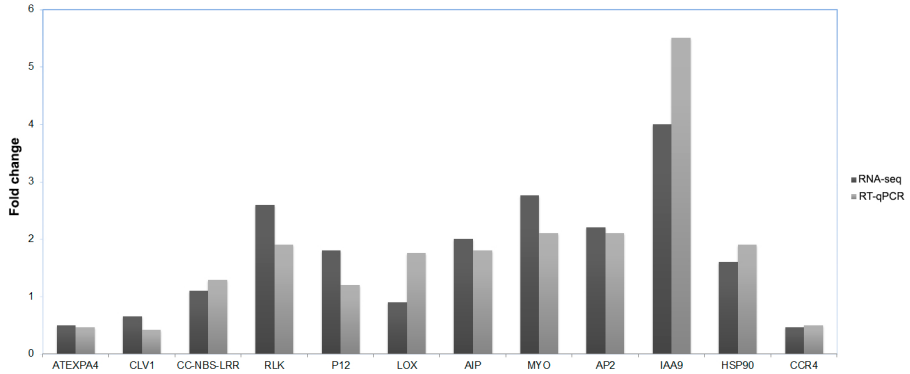

Supplement: Additional file 8 — Validation of 12 differentially expressed genes selected from RNA-seq analysis by RT-qPCR. The fold changes are shown for 12 differentially expressed genes identified using RNA-seq compared to those obtained by RT-qPCR. For this, cDNAs were prepared from RNA of Ponkan mandarin xylem tissue infected with X. fastidiosa or not (control) after one day, with three biological replicates. RT-qPCR data were normalized to the two most stable endogenous control genes (UBQ and CYP). [file 1471-2164-14-676-S8.pdf]

M-Value

0.06  
0.05  
0.04  
0.03  
0.02  
0.01  
0

UBQ

CYP

ETEF2

EGIDH

TUB

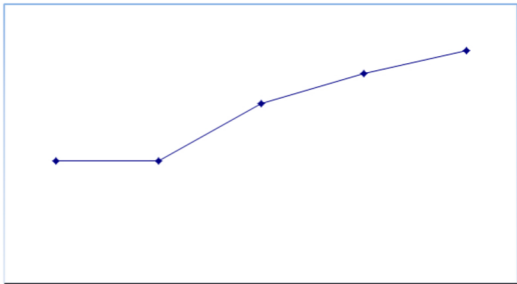

Supplement: Additional file 11 — Average expression stability values (M) of the five citrus endogenous control genes calculated by geNorm. Expression stability values were calculated for samples from citrus genotypes infected with X. fastidiosa and controls (mock). A lower M value indicates more stable expression. [file 1471-2164-14-676-S11.pdf]
