# Supplementary material for: RNA-Seq analysis of Citrus reticulata in the early stages of Xylella fastidiosa infection reveals auxin-related genes as a defense response
Source: BMC Genomics. 2013 Oct 3;14:676. doi: 10.1186/1471-2164-14-676 (PMC3852278; doi:10.1186/1471-2164-14-676)
Supplement: Additional file 10 — Validation of the specificity and amplification efficiency of the RT-qPCR primers. Amplification of cDNA for Ponkan mandarin genes ATEXPA4, CLV1, CC-NBS-LRR, LRR-RLK, P12, LOX, AIP, MYO, AP2, HSP90, CCR4, IAA9, ARF19, TIR1, BIG, E3, PR1 and CESA4. (A) Verification the dissociation pattern obtained after RT-qPCR. Each gene showed a single peak after melting curve analysis, confirming the specificity the primers. (B) Efficiency of amplification using primers obtained through Miner software after RT-qPCR. [file 1471-2164-14-676-S10.pdf]

A

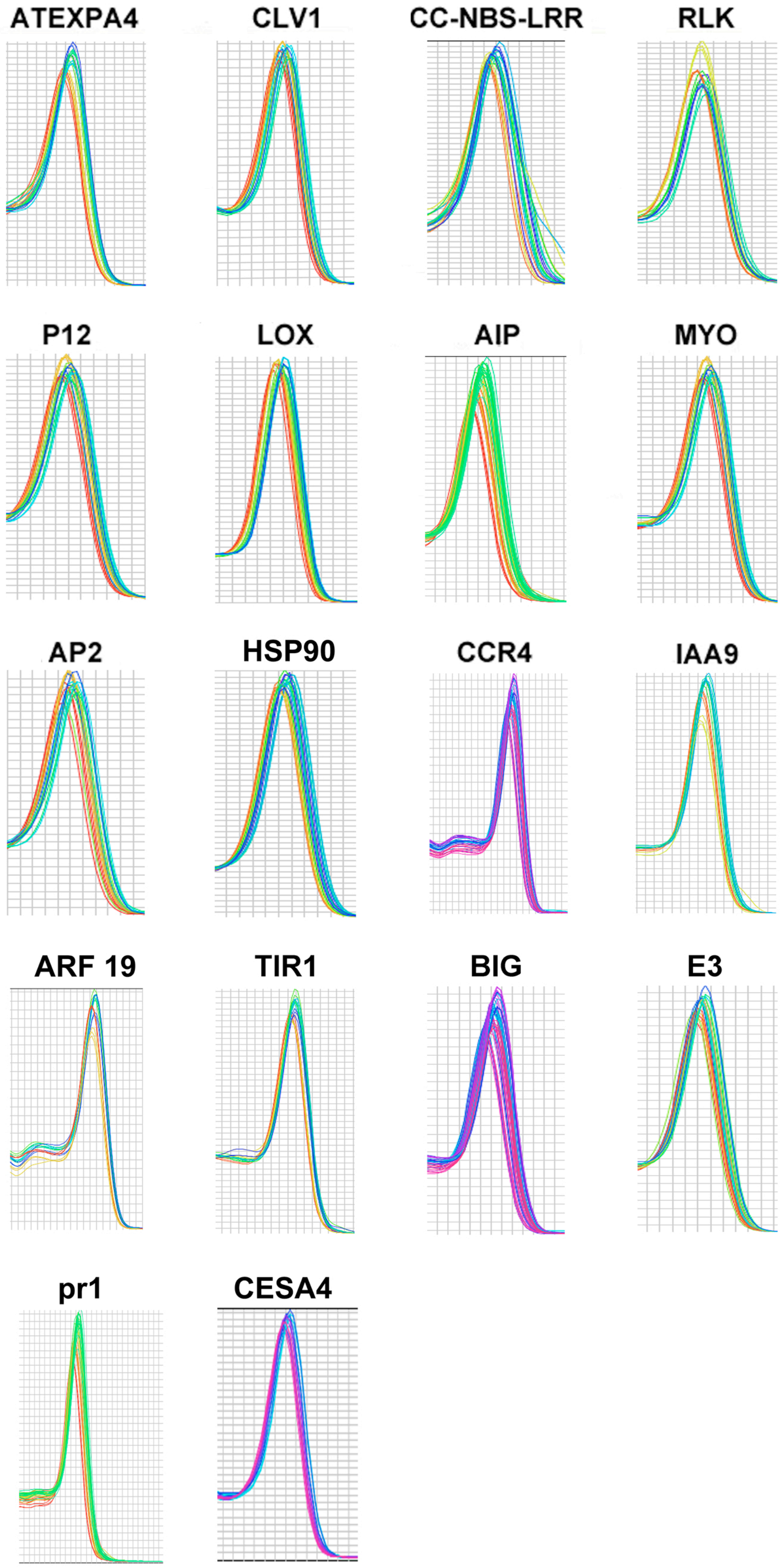

B

Gene	efficiency (%)
ATEXPA4	0.98
CLV1	1.00
CC-NBS-LRR	0.99
LRR-RLK	1.03
P12	1.01
LOX	0.97
AIP	1.01
MYO	0.99
AP2	1.06
HSP90	0.92
CCR4	0.99
IAA9	0.93
ARF19	1,00
TIR1	0.99
BIG	0.95
E3	0.99
pr1	0.98
CESA4	1.00
